# Curly fingernails as a potential new nail variant: A report of two cases

**DOI:** 10.1016/j.jdcr.2024.06.043

**Published:** 2024-08-22

**Authors:** Kaya L. Curtis, Jose W. Ricardo, Shari R. Lipner

**Affiliations:** a Weill Cornell Medical College, New York, New York; b Department of Dermatology, Weill Cornell Medicine, New York, New York

**Keywords:** curled nails, curly nails, curved nails, nail disorders

## Introduction

Curly nails are clinical finding in which the distal nail plate (NP) grows in an undulating and wavy fashion. Although it is a benign nail condition, it may be underreported and cause distress because of limited functionality and unsightly appearance. To our knowledge, only 1 case of curly fingernails has been reported to date by Oberlin et al.^1^ Here, we present 2 patients with curly fingernails who presented to our nail specialty clinic.

## Case reports

### Case 1

**Fig 1:**
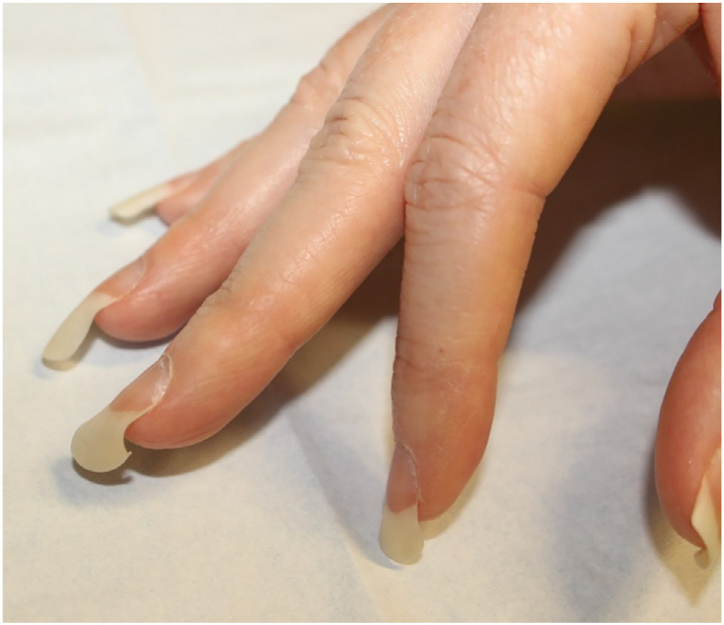
A 40-year-old woman patient with curly fingernails of the right hand.

**Fig 2:**
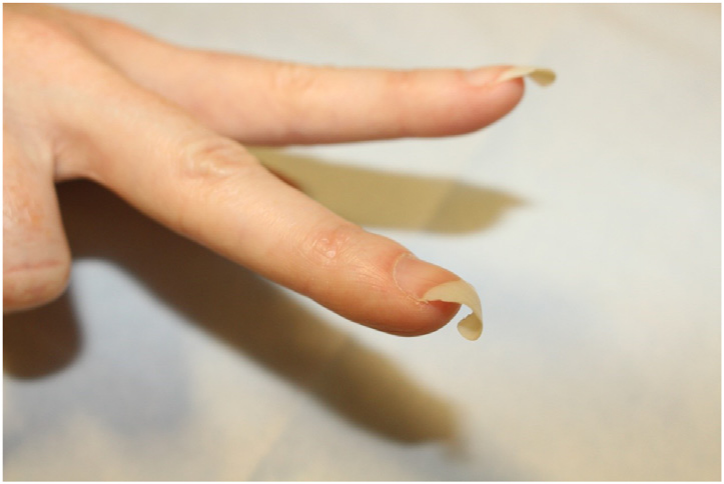
A 40-year-old woman patient with wavy and curly fingernails of the left hand.

A 40-year-old woman undergoing *in vitro* fertilization presented with a 30-year history of curly fingernails. She reported keeping her nails long regularly and noted that the curling was less notable when nails were short. She denied family history of similar nails, frequent use of hands for work or hobbies, or toenail involvement. She had a childhood history of right third digit trauma and a history of osteoarthritis. On physical examination, the fingernails were long and curled with minimal onycholysis (Figs 1 and 2). Toenails were normal. Histopathology of fingernail clippings were negative for presence of hyphae and parakeratosis with neutrophil infiltration, making diagnoses of onychomycosis and nail psoriasis unlikely. Bilateral hand X-rays were negative for psoriatic arthritis. She had curly hair. She reported a temporary episode of hair loss that coincided with her *in vitro* fertilization cycle, which resolved without treatment. At her 2-year follow-up, she reported that her nails were status quo and that she kept them short to minimize curliness.

### Case 2

**Fig 3:**
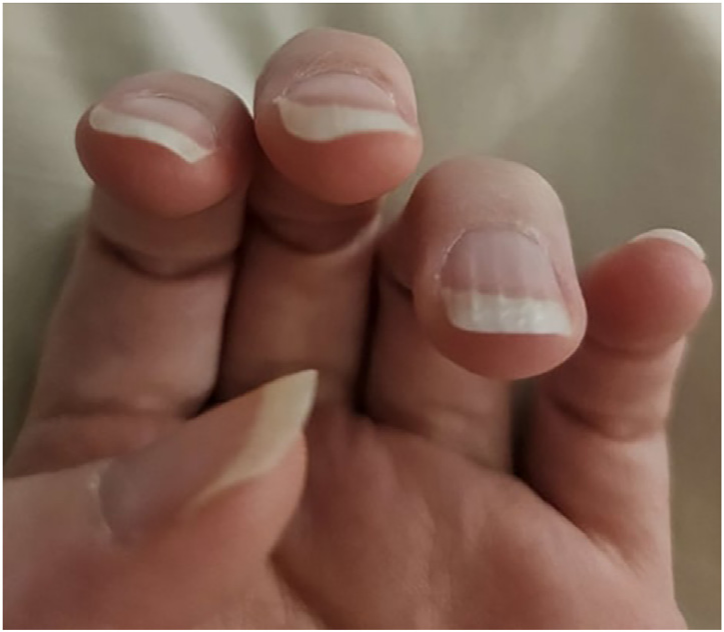
A 38-year-old woman patient, with curly fingernails of the left hand.

A 38-year-old woman, 34 weeks pregnant, presented with curved and wavy nails. She reported that her fingernails began growing curved and wavy at 30 to 31 weeks of pregnancy. She recalled a brief episode of curly nails in the remote past. She complained that the curved areas of the nails were painful, which was relieved by cutting the nails short. She described her hair as wavy. On physical examination, her fingernails were undulating with minimal onycholysis (Fig 3).

## Discussion

To our knowledge, only 1 case of curling of the fingernails has been reported, by Oberlin et al,^1^ of a 55-year-old African American woman with extremely long (lengths 5-30 cm) and curly fingernails. The primary differential diagnosis is parrot beak nails, characterized by excessive forward NP curvature of the free margin, and associated with trauma and systemic sclerosis. However, the overcurvature associated with parrot beak nails causes skin impingement and pain, whereas curly nails grow outward in an undulating fashion. Cohen^2^ reported a case of curly cuticles, but not NP, in an 83-year-old male patient, and proposed the term “eponychogryphosis” for this condition.

Congenital curved nail of the fourth toe, a rare disorder in which the fourth toe’s NP curves toward the plantar foot,^3^ was first reported in 1991. Since then, only 19 cases have been reported,^3^ most of them in Japan, with only one case outside Asia. Most cases had bilateral involvement (15/19), with pain in 8 of 19 cases, and men affected in 16 of 19 cases.^3^ This nail disorder is thought to develop because of terminal phalanx shortening and soft tissue hypoplasia of the fourth toe. Etiology is hypothesized to be a mesodermal abnormality, as an ectodermic abnormality would result in involvement of multiple toes or fingers.^4^ X-ray frequently demonstrates isolated distal phalanx shortening of the fourth toe(s). Several cases had a positive family history, suggesting a potential genetic mutation. Involvement of all fingernails in our and Oberlin et al’s^1^ cases suggest a pathogenesis distinct from congenital curved nail of the fourth toe, although the 2 phenomena may be related.

Genetic mutations causing curved nails have been reported in several mouse models. Mice homozygous for mutations in the hairless gene (*Hr*) develop universal hair loss and long, curved nails early in life.^5^ Mutations in the human hairless gene are responsible for congenital hair disorders, including congenital alopecia universalis. Although trachyonychia may be observed in association with congenital alopecia universalis,^6^ curved nails in these patients have been rarely reported.^7^

Sundberg et al^5^ reported on a spontaneous, autosomal, recessive mutation called witch nails (*whnl*) in a novel keratin gene, *KRT90*, resulting in development of excessively long and curled nails in mice. Although human *KRT90P* (keratin 90 pseudogene) is not expressed in the human nail unit, this mouse model finding may serve as a tool to further understand the role of conserved sequences of unknown function of certain keratins in normal nail growth patterns.^5^ Interestingly, a translational frame shift in *KRT5* is responsible for a rare form of epidermolysis bullosa simplex,^8^ whereas a frame shift in *KRT1* results in a rare type of ichthyosis.^9^ Expression of *KRT90* in the hair shaft and epidermal corneocytes in mice has not been studied. Histologically, there is extension of cornified hyponychium under the NP resulting in separation of the NP from the nail bed in abnormal nails because of mutations in *Hr* and *KRT90.*^5^

Oberlin et al^1^ hypothesized that ethnic differences might influence nail shape, analogous to hair, with asymmetric hair follicle differentiation in curly-haired Black patients and uniform differentiation in straight-haired individuals. For instance, Guinness World Record holders for longest fingernails have been a Caucasian woman with straight nails, and African American and Indian men with curly nails.^10^ Nonetheless, current hair classifications by geo-racial distinctions do not fully reflect the global diversity or cross-group overlap of hair types. Differences in nail manicuring techniques, as well as lipid and protein nail content, may also explain differences in nail morphology.

Limitations include lack of laboratory evaluation and genetic testing.

Curly fingernails are a rarely reported phenomenon with unknown pathogenesis. This novel clinical finding may serve as a window into previously unrecognized differences in nail morphologies across population groups or potential mutations in keratin genes related to other cutaneous diseases. However, such etiologies are only speculative and further characterization of this phenomenon is needed. Future studies are needed to assess the prevalence of curly nails, and histopathologic examination of this new nail variant may be enlightening. Unique cultural and psychosocial practices surrounding nails adds an extra layer of mystery in our understanding of this skin appendage.

## Conflicts of interest

Dr Lipner has served as a consultant for Ortho Dermatologics, Eli Lilly, BelleTorus Corporation, and Moberg Pharmaceuticals. Dr Ricardo and author Curtis have no conflicts of interest to declare.
